# New Perspectives on Gluten-Free Diet

**DOI:** 10.3390/nu12113540

**Published:** 2020-11-18

**Authors:** Paolo Usai-Satta, Mariantonia Lai

**Affiliations:** 1 Gastroenterology Unit, Brotzu Hospital, 09124 Cagliari, Italy; 2 Gastroenterology Unit, University of Cagliari, 09042 Monserrato, Italy; laimariantonia@gmail.com

Celiac disease (CD) is a permanent, chronic, gluten-sensitive disorder characterized by small intestinal inflammation and malabsorption in genetically predisposed individuals [1]. In addition, a self-reported gluten/wheat sensitivity without the diagnostic features of CD has recently been named non-celiac gluten/wheat sensitivity (NCGWS) [2].

The only effective and safe treatment for CD and gluten-related disorders (GRD) is a lifelong, strict exclusion of gluten, the so-called gluten-free diet (GFD). In this respect, there are new concepts and perspectives regarding GFD and its impact on clinical practice.

This Special Issue, entitled “Advance in Gluten-Free Diet”, comprises eight peer-reviewed papers reporting on different points of view regarding GFD in different clinical conditions.

In detail, the interplay between irritable bowel syndrome (IBS) and GRD, the role of GFD compared to low fermentable oligo/di/monosaccharides and polyols (FODMAP) diet (LFD) in IBS and functional dyspepsia (FD), the role of a low nickel diet in CD on GFD with persistent IBS-like symptoms, the efficacy of high-iron diet in CD with iron deficiency without anemia, the current reformulation of gluten-free food composition in Spain, the nutritional value of GFD in Polish CD prisoners and the symptoms worsening after wheat ingestion in familial Mediterranean fever are discussed in this Special Issue.

IBS is frequently associated with CD, and IBS symptoms may also overlap and be similar to those associated with NCGWS. In addition, many patients with CD have persistent digestive symptoms despite a strict GFD. This can be due to a higher frequency of IBS in CD patients compared to the general population. On the other hand, many different dietary approaches have been recently suggested for IBS and a GFD is considered a therapeutic option in a subset of IBS patients [3].

In their review, Bellini et al. [4] discuss the evidence regarding two of the most advised diets for IBS, the GFD and the LFD. A GFD is less restrictive and easer to follow than LFD. On the other hand, according to recent evidence, LFD is the most effective dietary intervention suggested for treating IBS, and it is included in the most updated guidelines. Unfortunately, the clinical trials regarding the dietary intervention for IBS are of low quality. The problem is the difficulty in setting up randomized double-blind controlled trials which objectively evaluate clinical results without the risk of a nocebo/placebo effect.

Similarly to IBS, both GFD and LFD could improve symptoms in patients with FD. In a double-blind, randomized, placebo controlled pilot trial, Potter et al. [5] have evaluated the role of this diet (specifically gluten and fructan) in patients with FD. A combined GFD–LFD led to an overall improvement in dyspeptic symptoms but this result was not significant. Otherwise, a specific food trigger was not identified. The authors consequently suggest further larger studies to confirm these data.

As hypothesized by Borghini et al. [6], a nickel-rich diet could exacerbate or relapse IBS-like symptoms in CD patients on strict GFD. In fact, many gluten-free foods are high in nickel content. In their study, 20 celiac patients on GFD, with persistent digestive symptoms and with positive patch test for nickel-mucositis, consumed a low-nickel diet. The result was an overall improvement in digestive symptoms in CD patients, with significant effects for 10 out of 24 symptoms (according to Gastrointestinal Symptom Rating Scale modified questionnaire). The impact of a nickel-rich diet on CD could be a clinical and scientific challenge for further studies to address.

Iron deficiency without anemia is a common clinical scenario in CD despite a strict GFD. A recommended approach to this condition is not yet defined. Scricciolo et al. [7] have compared a 12-week iron-rich diet to iron supplementation with ferrous sulfate in 22 celiac adult women. At the end of the treatments, both well tolerated, ferritin levels were statistically higher in the ferrous sulfate group. An iron-rich diet can be, however, recommended in patients who do not tolerate pharmacological supplementation.

The objective of the paper by Fajardo et al. [8] was to develop a nutritional food composition database including cereal-based gluten-free products available in Spain. A comprehensive database of 629 products was achieved. Gluten-free products were primarily composed of rice and/or corn flour. The most common added fat was sunflower oil, followed by palm fat, olive oil and cocoa. Xanthan gum was the most frequently employed fiber. Nutritional deficiencies have been described for CD patients on GFD and an updated quality assessment of available products is needed for further improvement in gluten-free product development.

A special clinical setting for CD patients can be represented by the prison population. The risk of nutritional deficiencies may be a real problem for CD prisoners due to the limited possibilities of external quality control. In the study by Kosendiak et al. [9], the nutritional value of GFD and regular diet meals served in 10 Polish prisons were assessed. GFD was characterized by lower average energy content in 11 out 14 essential nutrients. Greater quality control of GFD meals served in catering facilities should be recommended.

Finally, Carroccio et al. [10] have identified a clinical association between self-reported NCGWS and familial Mediterranean fever (FMF). In their pilot randomized trial, the authors have evaluated clinical and innate immune responses to wheat (compared to rice) challenge. In six NCGWS/FMF female patients, wheat ingestion exacerbated clinical and immunological features of FMF. These findings may suggest new clinical scenarios in the management of FMF.

In conclusion, the different perspectives presented in this Special Issue confirm that the gluten-free diet is currently a clinically and scientifically challenging topic. We would like to thank all the authors and the editorial team of *Nutrients* for their precious contributions.
